# Heterogeneous trajectories of nutritional support needs in patient–caregiver dyads following radical esophagectomy: a latent class growth analysis

**DOI:** 10.3389/fnut.2026.1789423

**Published:** 2026-05-14

**Authors:** Wei Guo, Jing Cui, Yiqian Ni, Yanhua Li, Bowen Shi, Xue Liu, Wenqian Qi, Yi Zhang

**Affiliations:** 1 School of Nursing, Naval Medical University, Shanghai, China; 2 The First Affiliated Hospital of Naval Medical University, Shanghai, China; 3 The First Affiliated Hospital of Zhengzhou University, Henan, China; 4 Eye & ENT Hospital, Fudan University, Shanghai, China; 5 Department of Cardiovascular Medicine, the 940th Hospital of Joint Logistic Support Force, Lanzhou, China

**Keywords:** latent class growth analysis, latent growth curve model, malnutrition, nutritional support needs, patient and family caregiver

## Abstract

**Introduction:**

Patients with esophageal cancer are at high risk of malnutrition, with family caregivers playing an indispensable role in nutritional management. However, the dynamic and heterogeneous changes of nutritional support needs of patients after radical esophagectomy, viewed from the perspectives of both patients and their caregivers, remain poorly understood. This may potentially hinder the development of timely and tailored interventions. Therefore, this study aimed to investigate the longitudinal trajectories of nutritional support needs within patient–caregiver dyads and identify distinct patterns of change over time, and explore associated influencing factors.

**Methods:**

This prospective longitudinal study recruited 159 patient–caregiver dyads from a tertiary hospital in Shanghai, China, between August 2024 and January 2025. Data were collected at four time points: pre-discharge (T1), 2 weeks (T2) post-discharge, 1 month (T3) post-discharge, and 3 months (T4) post-discharge. The Questionnaire of Nutritional Needs in Patients after Esophagectomy (QNN-E) was used to assess dyadic nutritional support needs. Trajectory homogeneity and heterogeneity were analyzed using latent growth curve modeling and latent class growth analysis. Baseline factors, including General Self-Efficacy Scale (GSES), quality of life (QLQ-OES18), Hospital Anxiety and Depression Scale (HADS), Activities of Daily Living (ADL), and Patient-Generated Subjective Global Assessment (PG-SGA), were examined as predictors of trajectory membership.

**Results:**

The nutritional support needs of patient–caregiver dyads declined over time. Significant heterogeneity was observed in therapeutic diet preparation, symptom surveillance and complication management. For therapeutic diet preparation, two distinct trajectories emerged in the patients (*Consistently Stable Needs* [16.4%] and *Delayed Needs Escalation* [83.6%]), influenced by enteral access type and nutritional status. The trajectories of the family caregivers (*High-need Declining* [47.2%] and *High-need Sustenance* [52.8%]) were associated with educational level and relationship to the patient. In symptom surveillance and complication management, the trajectories of the patients were influenced by basic activities of daily living, while the trajectories of the caregivers were linked to the presence of other caregivers.

**Conclusion:**

The findings revealed significant heterogeneity in the postoperative nutritional support needs of patient-caregiver dyads, influenced by distinct factors. As primary providers of postoperative home-based nutritional care, caregivers’ needs provide valuable insights for a more comprehensive understanding of postoperative nutritional care requirements. Continuity of nutritional care for patients with esophageal cancer should be grounded in dynamic and holistic assessments of their support needs, enabling personalized interventions at critical time points to improve postoperative recovery outcomes and care effectiveness.

## 1 Introduction

Esophageal cancer is associated with the highest incidence of malnutrition among all solid tumors and significantly affects patients’ nutritional status, postoperative recovery, and quality of life (QoL) (1, 2). Radical esophagectomy, including gastric and colonic interposition, remains the primary curative treatment (3). Preoperative malnutrition often results from dysphagia and tumor-induced obstruction, while postoperative nutritional deterioration is exacerbated by surgical stress and anatomical reconstruction (4). Notably, malnutrition prevalence is even higher among patients after esophagectomy post-discharge (60–85%) than during hospitalization (5, 6).

Postoperatively, nutritional support typically initiates with tube feeding via nasojejunal or jejunostomy, often continuing as home enteral nutrition before gradually transitioning to oral intake as healing progresses (7, 8). Consequently, nutritional support needs of patients with esophageal cancer evolve dynamically across the care continuum, influenced by changes in nutritional intake methods (9). Considering the temporal complexity, cross-sectional assessments are insufficient to capture dynamic trajectory of nutritional support requirements. Longitudinal investigations are therefore essential to delineate how nutritional support needs evolve across the postoperative trajectory. A one-year prospective study of postoperative patients identified distinct needs at different stages: early-phase needs focused on food and nutrient selection, while later phases involved adapting to new dietary habits (10). Grace et al. (11) reported that malnutrition either persisted or worsened in most patients over 12 months and was associated with inadequate dietary intake. However, a mismatch between the nutritional support provided and patients’ actual needs often delays interventions and causes adverse clinical outcomes (12). Therefore, the actual nutritional support needs of patients must be explored to provide appropriate interventions.

Furthermore, as the patients frequently experience limited self-care capacity during recovery, family caregivers play an indispensable role in postoperative nutritional management. Their support needs often reflect patients’ actual needs during nutritional management, serving as a proxy for unmet patient needs within the caregiving context (13). Although nutritional needs are inherently dynamic, most existing evidence is derived from cross-sectional studies or focuses exclusively on patients, failing to capture the longitudinal evolution and dyadic nature of these needs within the patient-caregiver unit.

This highlights a significant knowledge gap. Existing longitudinal studies have largely examined patients or caregivers in isolation, employing a unidimensional perspective that overlooks the inherently interdependent nature of post-discharge recovery. Within the patient-caregiver dyad, the caregiver’s evolving needs and capacities are both shaped by and, in turn, shape the patient’s condition. Consequently, there is a paucity of knowledge regarding the distinct, yet potentially interrelated, longitudinal trajectories of nutritional support needs within these dyads and the factors that determine their evolution. A detailed understanding of these co-evolving trajectories is crucial to inform the development of targeted, timely, and dyad-centered interventions. To address this gap, this prospective longitudinal study aimed to (1) model the heterogeneous trajectories of nutritional support needs in patients and their family caregivers during the first 3 months following esophagectomy, and (2) identify the key factors associated with different trajectory patterns.

## 2 Methods

### 2.1 Design and participants

This prospective longitudinal study was conducted in the Department of Thoracic Surgery at a tertiary hospital in Shanghai, China, between August 2024 and January 2025. Patient–caregiver dyads were enrolled only if both parties provided written informed consent. Eligible patients met the following criteria: (1) diagnosis of esophageal cancer according to the *Chinese Guidelines for Diagnosis and Treatment of Esophageal Cancer (2020 Edition)* (14); (2) having undergone radical esophagectomy; (3) receiving active home-based enteral nutrition support; (4) exhibiting nutritional risk (Nutritional Risk Screening 2002 [NRS 2002] score ≥ 3) (15); (5) age >18 years; and (6) Mandarin proficiency. Exclusion criteria included severe postoperative complications (e.g., anastomotic leakage, sepsis), neuropsychiatric disorders impairing communication, or inability to complete follow-up assessments. Family caregivers were required to be (1) age >18 years, (2) fluent in Mandarin, and (3) designated as the primary unpaid family caregiver (providing direct care ≥4 h/day). Those professionally engaged in nutrition-related occupations and non-family members (e.g., hired nurses) were excluded. All questionnaires were administered and collected immediately after completion. Participants were assured of data confidentiality (anonymized storage and analysis), the right to withdraw at any time without penalty, and exclusive use of data for research purposes. Based on previous findings (16), disease characteristics, and clinical guideline recommendations (17), four assessment timepoints were established: pre-discharge (T1), 2 weeks post-discharge (T2), 1 month post-discharge (T3), and 3 months post-discharge (T4). Sample size estimation was guided by statistical tables for a single-group repeated-measures design (18), accounting for four assessment points. The initial calculation yielded 142 dyads, expanded to 156 (10% increase) to accommodate potential attrition. The study was approved by the Committee on Ethics of Medical Research at Naval Medical University (Supplement 1), which adhered to the Strengthening the Reporting of Observational Studies in Epidemiology (STROBE) reporting guidelines.

### 2.2 Measures

Patient assessments included seven validated instruments: a demographic information questionnaire, the Questionnaire of Nutritional Needs in Patients after Esophagectomy (QNN-E), the Chinese General Self-Efficacy Scale (C-GSES), the Chinese Quality of Life Questionnaire–Oesophageal Cancer Module (C-QLQ-OES18), the Chinese Hospital Anxiety and Depression Scale (C-HADS, integrating subscales for anxiety [C-SAS] and depression [C-SDS]), the Chinese Activities of Daily Living Scale (C-ADL, comprising basic [C-BADL] and instrumental [C-IADL] domains), and the Chinese Patient-Generated Subjective Global Assessment (C-PG-SGA). Family caregiver completed three instruments: a demographic questionnaire, the QNN-E, and the C-GSES. The QNN-E was administered longitudinally to both patients and caregivers at four timepoints (T1–T4). All other instruments were administered only at baseline (T1).

#### 2.2.1 Demographic information questionnaire

Demographic characteristics of patients and family caregivers were collected, including gender, age, educational level, occupation, discharge destination, enteral access type, monthly income, relationship to patients, and other caregivers.

#### 2.2.2 The questionnaire of nutritional needs in patients after Esophagectomy

The QNN-E is a 49-item self-report instrument developed to assess nutritional support needs in patient–caregiver dyads following radical esophagectomy (19). The development included qualitative interviews, literature reviews, and a two-round Delphi consensus process to ensure content validity. Items are rated on a 5-point Likert scale (1 = not at all needed to 5 = completely needed) across five domains. The QNN-E demonstrates excellent psychometric properties, with a total Cronbach’s *α* of 0.965, subscale *α* values ranging from 0.895 to 0.945, and a content validity index of 0.995. Total scores are calculated by summing all items, with higher scores indicating greater perceived nutritional support needs.

The five dimensions are as follows: (1) Enteral Access Device Management (12 items), assessing competence in maintaining and operating enteral feeding tubes, including cleaning protocols, blockage resolution, and prevention of displacement; (2) Therapeutic Diet Preparation (12 items), focusing on personalized meal planning and texture modification during the post-discharge transition; (3) Symptom Surveillance and Complication Management (13 items), evaluating the ability to identify and address critical complications and discomfort; (4) Longitudinal Nutritional Counseling (7 items), capturing the need for ongoing professional nutritional guidance; and (5) Adaptive Living Strategies (5 items) addressing psychosocial and functional adjustments for holistic recovery.

#### 2.2.3 General self-efficacy

The GSES is a 10-item self-administered instrument measuring optimistic self-beliefs regarding coping competence across diverse life demands and challenges (20). The Chinese version (C-GSES) (21) employs a 4-point Likert-type scale (1 = not at all correct; 4 = completely correct), yielding total scores ranging from 10 to 40. Higher values indicate stronger perceived self-efficacy. The Cronbach’s *α* was 0.870 for patients and 0.832 for caregivers.

#### 2.2.4 Quality of life

The C-QLQ-OES18 is a disease-specific 18-item instrument assessing QoL across 10 domains: pain, eating, dysphagia, reflux, choking, swallowing obstruction, dry mouth, appetite loss, coughing, and speech problems. Items are rated on a 4-point Likert-type scale (1 = “not at all”; 4 = “very much”) (22). Higher scores indicate greater symptom burden or functional impairment (except for dysphagia, for which higher scores reflect milder symptoms). The instrument has demonstrated robust psychometric properties in esophageal cancer populations (23), with a Cronbach’s *α* of 0.718.

#### 2.2.5 Anxiety and depression

The HADS is a 14-item screening tool for anxiety and depression. It comprises two 7-item subscales (Anxiety: items 1, 3, 5, 7, 9, 11, and 13; Depression: items 2, 4, 6, 8, 10, 12, and 14) rated on a 4-point Likert-type scale (0 = “not present” to 3 = “severe”) (24). Subscale scores range from 0 to 21, and scores ≥8 indicating clinically significant symptoms. The C-HADS exhibits a sensitivity and specificity of >90% in esophageal cancer populations (25), with a Cronbach’s alpha of 0.885.

#### 2.2.6 Activities of daily living

The ADL is a 14-item instrument measuring functional independence across basic self-care (BADL: 6 items) and instrumental living skills (IADL: 8 items) (26). Items are rated on a 4-point Likert-type scale (1 = “performs independently” to 4 = “completely dependent”). Total scores range from 14 to 56, with scores >14 indicating functional decline. Subscale-specific thresholds identify impairment (BADL > 6; IADL > 8). The Chinese version demonstrates robust psychometric properties in oncology populations (27, 28), with a Cronbach’s *α* of 0.734.

#### 2.2.7 Malnutrition assessment

The PG-SGA is a malnutrition screening instrument that combines patient self-report (weight, dietary intake, symptoms, and functional capacity) and clinician assessment (disease status, metabolic stress, and physical signs) (29). Total scores categorize nutritional status as well-nourished (0–1), moderate malnutrition (2–8), or severe malnutrition (≥9). Endorsed by the Chinese Oncology Nutrition Guidelines (Class I evidence), the Chinese version correlates with functional performance scales in cancer populations (30).

### 2.3 Statistical analysis

Data were analyzed using IBM SPSS v26.0 and Mplus version 8.3. Descriptive analyses were performed to characterize the sample. Categorical variables were calculated as frequencies and percentages, and continuous variables as means ± standard deviations (SDs). A latent growth curve model (LGCM) and latent class growth analysis (LCGA) were used to explore the developmental trajectories of nutritional support needs within patient–caregiver dyads.

LGCM was used to characterize longitudinal changes in variables by separating the actual variance in the model from the measurement error and enabling estimations of the actual change in latent variables over time (31). Model fit was evaluated using *χ*^2^/degrees of freedom (*df*) ratio, root mean square error of approximation (RMSEA), standardized root mean square residual (SRMR), and comparative fit index (CFI). The dyads’ developmental trajectories of the nutritional support needs were identified using unconditional free estimation. The intercept represents the level of nutritional support needs at each stage and the slope represents the rate of change in nutritional support needs.

LCGA was employed to identify distinct subgroups of dyads with similar trajectory patterns based on individual response profile (32). The optimal number of latent classes was determined by evaluating model fit indices, including the Akaike Information Criterion (AIC), Bayesian Information Criterion (BIC), and sample size-adjusted BIC (aBIC), with lower values indicating superior fit. Classification accuracy was assessed using entropy (range 0–1, values closer to 1 preferred). The likelihood ratio test (LRT) and bootstrap likelihood ratio test (BLRT) were used for model comparison; a significant *p-*value (<0.05) for the *k*-class model suggested it was superior to the *k-1*-class model. Both statistical criteria (e.g., class probability >5%) and substantive interpretability were considered when finalizing the number of classes (33). Unconditional models were constructed using all QNN-E dimension scores as observed variables to examine the trajectories of dyads’ nutritional support needs. Subsequently, univariate analyses (independent samples *t-*tests and chi-square tests) were conducted to examine differences in demographic and clinical characteristics across the identified trajectory classes. Binary logistic regression was then used to explore associations between these significant variables and trajectory class membership. A *p*-value < 0.05 was considered statistically significant for all analyses.

## 3 Results

### 3.1 Sample characteristics

**Figure 1:**
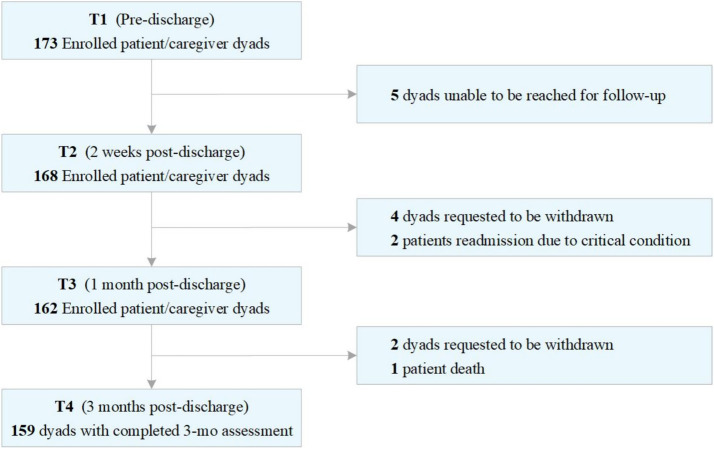
Participant enrollment.

The study enrolled 159 patient–caregiver dyads finally (Figure 1). The mean age of patients was 66.59 years (SD = 6.94), with a majority being male (78.62%). And the mean age of family caregivers was 46.86 years (SD = 8.56), with a majority being male (59.12%). Only 16.98% of patients and 65.41% of caregivers had an educational level of senior high school or above. Most of the patients (65.41%) were discharged with nasojejunal tubes. Regarding patient–caregiver relationships, 16.98% were spouses, 69.81% were adult children of patients, and 13.21% were others; 74.84% had additional family caregivers. The characteristics are detailed in Tables 1, 2.

**Table 1:** Baseline characteristics and inter-subgroup comparisons across dual nutritional trajectory dimensions in patients (*N* = 159).

Characteristic	Total sample, M ± SDs/No. (%) (*N* = 159)	Therapeutic diet preparation				Symptom surveillance and complication management			
		Class1-1: consistently stable needs (*N* = 26)	Class1-2: delayed needs escalation (*N* = 133)	*χ^2^*	*p*	Class2-1: persistently declining needs (*N* = 144)	Class2-2: gradually escalating Needs (*N* = 15)	*χ^2^*	*p*
Gender									
Men	125 (78.62)	19 (73.08)	106 (79.70)	0.57	0.451	116 (80.56)	9 (60.00)	3.41	0.065
Women	34 (21.38)	7 (26.92)	27 (20.30)			28 (19.44)	6 (40.00)		
Age, y	66.59 ± 6.94								
≤60	34 (21.38)	6 (23.08)	28 (21.05)	0.30	0.862	32 (22.22)	2 (13.33)	0.89	0.641
61–70	75 (47.17)	13 (50.00)	62 (46.62)			68 (47.22)	7 (46.67)		
≥71	50 (31.45)	7 (26.92)	43 (32.33)			44 (30.56)	6 (40.00)		
Education level									
Elementary and/or below	77 (48.43)	17 (65.38)	60 (45.11)	4.13	0.247	69 (47.92)	8 (53.33)	1.36	0.715
Junior high school	55 (34.59)	6 (23.08)	49 (36.84)			50 (34.72)	5 (33.33)		
Senior high school	22 (13.84)	3 (11.54)	19 (14.29)			21 (14.58)	1 (6.67)		
College and/or above	5 (3.14)	0 (0.00)	5 (3.76)			4 (2.78)	1 (6.67)		
Occupation									
Employed	27 (16.98)	6 (23.08)	21 (15.79)	0.82	0.365	25 (17.36)	2 (13.33)	0.16	0.693
Unemployed/retired	132 (83.02)	20 (76.92)	112 (84.21)			119 (82.64)	13 (86.67)		
Length of stay (in weeks)									
1–2	70 (44.03)	10 (38.46)	60 (45.11)	0.39	0.532	62 (43.06)	8 (53.33)	0.58	0.445
>2	89 (55.97)	16 (61.54)	73 (54.89)			82 (56.94)	7 (46.67)		
Discharge destination									
Home	132 (83.02)	25 (96.15)	107 (80.45)	3.80	0.051	119 (82.64)	13 (86.67)	0.16	0.693
Medical institution	27 (16.98)	1 (3.85)	26 (19.55)			25 (17.36)	2 (13.33)		
Enteral access type									
Nasojejunal tube	104 (65.41)	23 (88.46)	81 (60.90)	7.30	0.007	94 (65.28)	10 (66.67)	0.01	0.914
Jejunostomy tube	55 (34.59)	3 (11.54)	52 (39.10)			50 (34.72)	5 (33.33)		
Pathological stage									
I	40 (25.16)	5 (19.23)	35 (26.32)	0.58	0.748	36 (25.00)	4 (26.67)	2.36	0.308
II	79 (49.69)	14 (53.85)	65 (48.87)			74 (51.39)	5 (33.33)		
III	40 (25.16)	7 (26.92)	33 (24.81)			34 (23.61)	6 (40.00)		
Tumor location									
Upper third	14 (8.81)	3 (11.54)	11 (8.27)	0.40	0.818	12 (8.33)	2 (13.33)	3.37	0.186
Middle third	90 (56.60)	15 (57.69)	75 (56.39)			79 (54.86)	11 (73.33)		
Lower third	55 (34.59)	8 (30.77)	47 (35.34)			53 (36.81)	2 (13.33)		
Surgical approach									
Minimally invasive Esophagectomy	105 (66.04)	17 (65.38)	88 (66.17)	0.01	0.939	93 (64.58)	12 (80.00)	1.44	0.230
Open esophagectomy	54 (33.96)	9 (34.62)	45 (33.83)			51 (35.42)	3 (20.00)		

**Table 2:** Baseline characteristics and inter-subgroup comparisons across dual nutritional trajectory dimensions in caregivers (*N* = 159).

Characteristic	Total sample, M ± SDs/No. (%) (*N* = 159)	Therapeutic diet preparation				Symptom surveillance and complication management			
		Class1a: High-need Declining (*N* = 75)	Class1b: High-need Sustenance (*N* = 84)	*χ^2^*	*p*	Class2a: Rapidly Declining High-need (*N* = 145)	Class2b: Gradually Increasing Low-need (*N* = 14)	*χ^2^*	*p*
Gender									
Men	94 (59.12)	43 (57.33)	51 (60.71)	0.19	0.665	88 (60.69)	6 (42.86)	1.68	0.195
Women	65 (40.88)	32 (42.67)	33 (39.29)			57 (39.31)	8 (57.14)		
Age, y	46.86 ± 8.56								
≤40	36 (22.64)	18 (24.00)	18 (21.43)	5.44	0.066	33 (22.76)	3 (21.43)	0.12	0.940
41–50	84 (52.83)	33 (44.00)	51 (60.71)			76 (52.41)	8 (57.14)		
≥51	39 (24.53)	24 (32.00)	15 (17.86)			36 (24.83)	3 (21.43)		
Education level									
Elementary and/or below	9 (5.66)	8 (10.67)	1 (1.19)	9.59	0.022	8 (5.52)	1 (7.14)	1.66	0.646
Junior high school	46 (28.93)	23 (30.67)	23 (27.38)			40 (27.59)	6 (42.86)		
Senior high school	72 (45.28)	27 (36.00)	45 (53.57)			67 (46.21)	5 (35.71)		
College and/or above	32 (20.13)	17 (22.67)	15 (17.86)			30 (20.69)	2 (14.29)		
Occupation									
Employed	131 (82.39)	58 (77.33)	73 (86.90)	2.50	0.114	120 (82.76)	11 (78.57)	0.15	0.694
Unemployed/retired	28 (17.61)	17 (22.67)	11 (13.10)			25 (17.24)	3 (21.43)		
Monthly income (RMB)									
<5,000	6 (3.77)	2 (2.67)	4 (4.76)	0.48	0.786	4 (2.76)	2 (14.26)	4.99	0.083
5,000–10,000	103 (64.78)	49 (65.33)	54 (64.29)			94 (64.83)	9 (64.29)		
>10,000	50 (31.45)	24 (32.00)	26 (30.95)			47 (32.41)	3 (21.43)		
Marital status									
Married	156 (98.11)	72 (96.00)	84 (100.00)	3.43	0.064	142 (97.93)	14 (100.00)	0.30	0.587
Unmarried	3 (1.89)	3 (4.00)	0 (0.00)			3 (2.07)	0 (0.00)		
Relationship with patient									
Spouse	27 (16.98)	18 (24.00)	9 (10.71)	10.49	0.005	25 (17.24)	2 (14.26)	0.66	0.718
Adult child	111 (69.81)	43 (57.33)	68 (80.95)			100 (68.97)	11 (78.57)		
Others	21 (13.21)	14 (18.67)	7 (8.33)			20 (13.79)	1 (7.14)		
Disease literacy level									
Understanding	2 (1.26)	0 (0.00)	2 (2.38)	2.91	0.233	2 (1.38)	0 (0.00)	0.30	0.863
Geneal	156 (98.11)	74 (98.67)	82 (97.62)			142 (97.93)	14 (100.00)		
Not understanding	1 (0.63)	1 (1.33)	0 (0.00)			1 (0.69)	0 (0.00)		
Other caregivers									
Yes	119 (74.84)	53 (70.67)	66 (78.57)	1.32	0.252	118 (81.38)	1 (7.14)	37.37	<0.001
No	40 (25.16)	22 (29.33)	18 (21.43)			27 (18.62)	13 (92.86)		

Family caregivers showed greater general self-efficacy (mean = 3.05, SD = 0.26) than patients (mean = 2.65, SD = 0.34) as measured using the C-GSES (Table 3). At baseline, patients reported relatively low scores across several health status indicators, including health-related QoL (mean = 1.90, SD = 1.90; C-QLQ-OES18), anxiety (mean = 1.06, SD = 0.22; C-SAS), depression (mean = 1.26, SD = 0.22; C-SDS), BADL (mean = 1.56, SD = 0.22; C-BADL), IADL (mean = 2.48, SD = 0.26; C-IADL), and nutritional status (mean = 11.05, SD = 1.96; C-PG-SGA).

**Table 3:** Baseline scores of the C-GSES, C-QLQ-OES18, C-HADS, C-ADL and C-PG-SGA for patient-caregiver.

Participant	Variable		Sum	Average
Patient (*N* = 159)	C-GSES		26.52 ± 3.42	2.65 ± 0.34
	C-QLQ-OES18		34.21 ± 3.17	1.90 ± 1.90
	C-HADS	C-SAS	7.41 ± 1.54	1.06 ± 0.22
		C-SDS	8.84 ± 1.56	1.26 ± 0.22
	C-ADL	C-BADL	9.34 ± 1.31	1.56 ± 0.22
		C-IADL	19.82 ± 2.04	2.48 ± 0.26
	C-PG-SGA		11.05 ± 1.96	2.76 ± 0.49
Caregiver (*N* = 159)	C-GSES		30.45 ± 2.63	3.05 ± 0.26

C-GSES, Chinese version of general self-efficacy scale; C-QLQ-OES18, Chinese version of quality of life questionnaire–oesophageal cancer module; C-HADS, Chinese version of hospital anxiety and depression scale; C-SAS, Chinese version of self-rating anxiety scale; C-SDS, Chinese version of self-rating depression scale; C-ADL, Chinese version of activities of daily living; C-BADL, Chinese version of basic activities of daily living; C-IADL, Chinese version of Instrumental activities of daily living; C-PG-SGA, Chinese version of patient-generated subjective global assessment.

### 3.2 Overall trajectory of nutritional support needs

**Figure 2:**
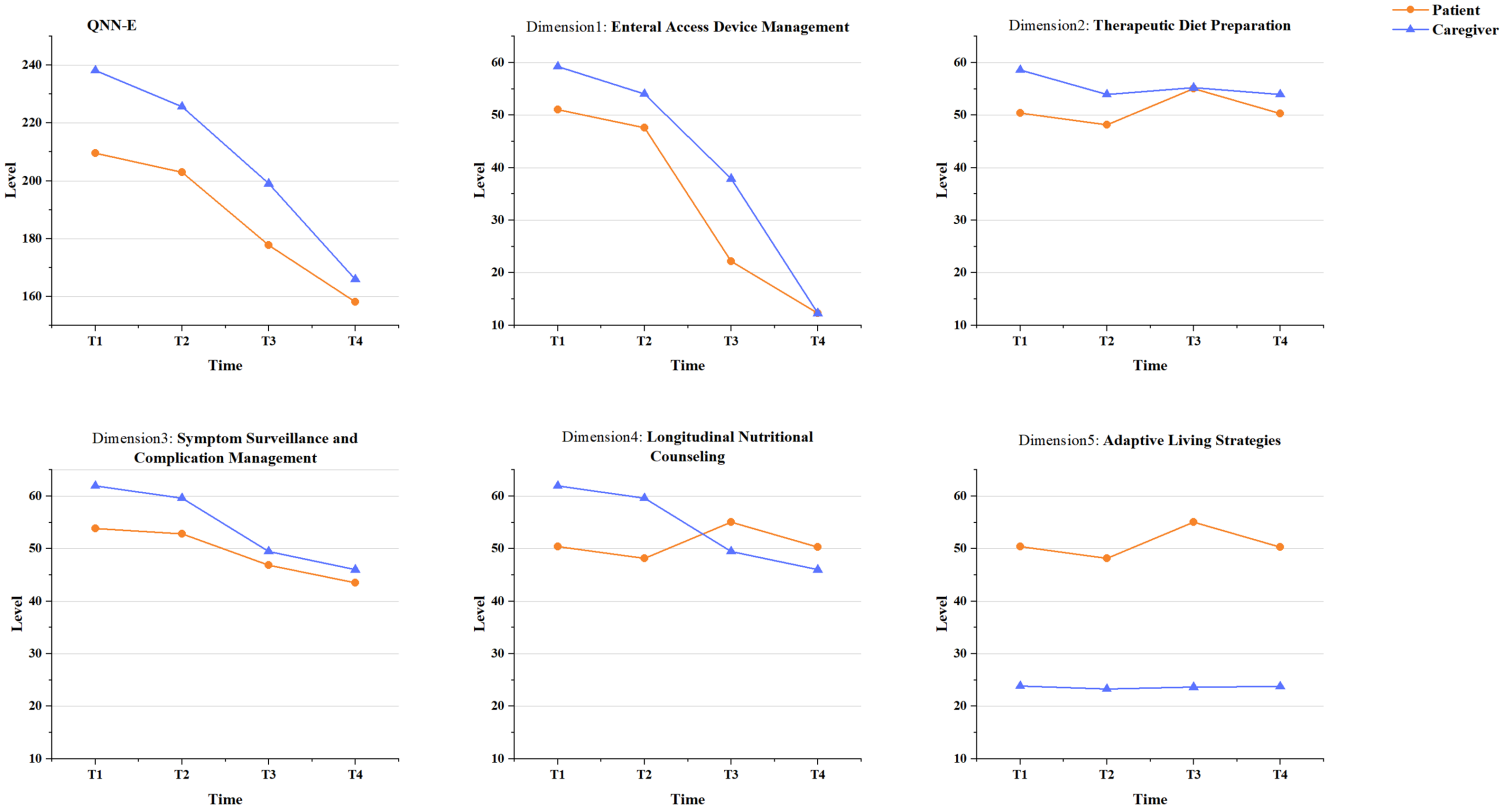
T1, Pre-discharge; T2, 2 weeks post-discharge; T3, 1 month post-discharge; T4, 3 months post-discharge; QNN-E, questionnaire of nutritional needs in patients after esophagectomy.

**Figure 3:**
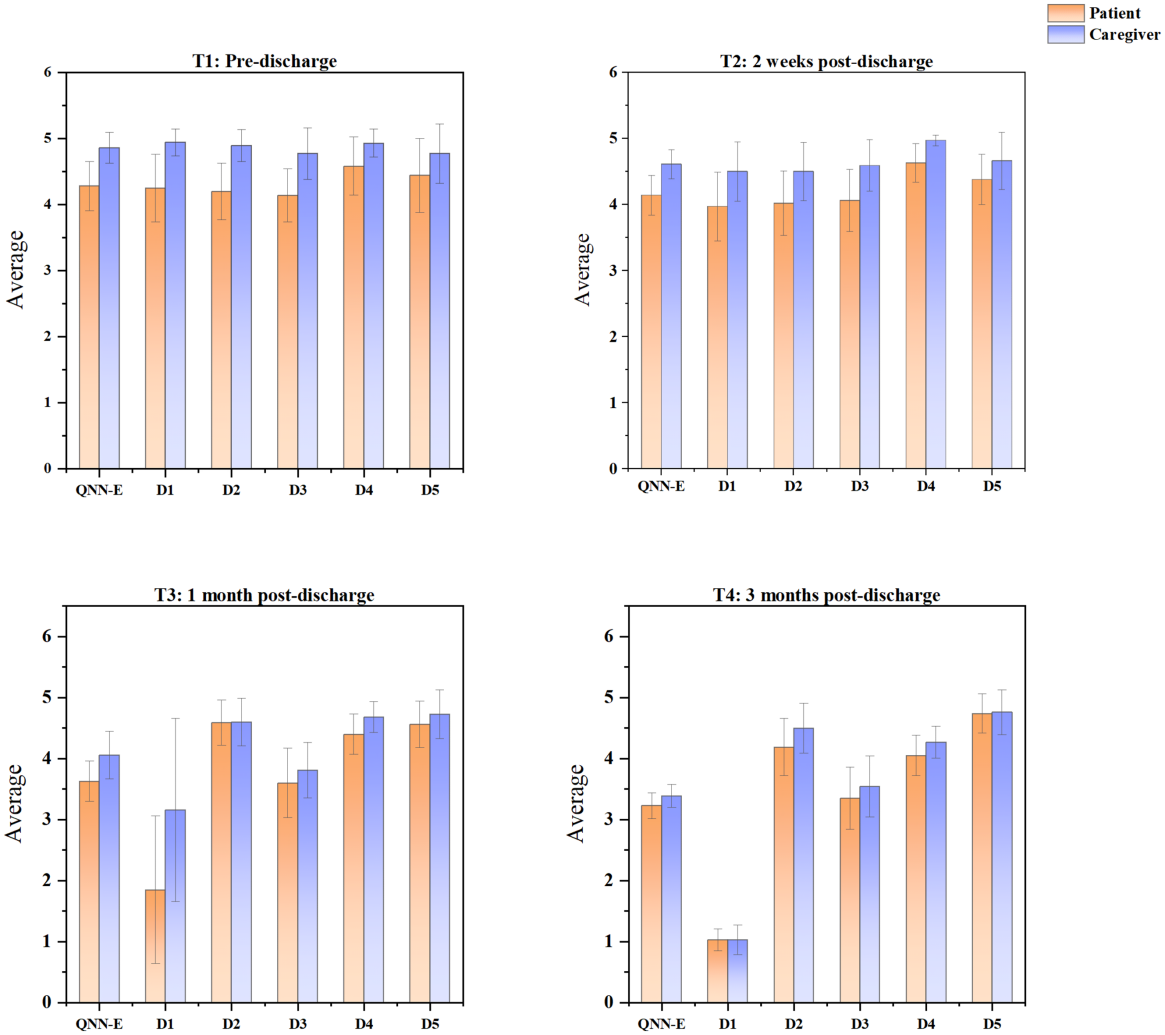
QNN-E, questionnaire of nutritional needs in patients after esophagectomy; D1, enteral access device management; D2, therapeutic diet preparation; D3, symptom surveillance and complication management; D4, longitudinal nutritional counseling; D5, adaptive living strategies.

The trajectories of nutritional support needs were revealed in patients and family caregivers from T1 to T4 by LGCM (Figure 2; complete model results are provided in Supplementary Table 1). Overall, the nutritional support needs for both patients and family caregivers decreased from T1 to T4. However, the family caregivers consistently reported higher levels of needs than patients across most domains and time points. Dimension-specific analyses revealed that, although patients and caregivers reported largely comparable levels of need in the *Therapeutic Diet Preparation* domain, with nearly equivalent scores observed at T3, distinct patterns emerged in other domains. Specifically, patients’ needs in *Longitudinal Nutritional Counseling* were lower than those of caregivers at T1 and T2, but surpassed them at T3 and T4. While patients consistently reported higher needs than caregivers in *Adaptive Living Strategies*. The QNN-E scores across the four time points are summarized in Table 4 and illustrated in Figure 3.

**Table 4:** QNN-E score dynamics: patient-caregiver comparisons across postoperative phases (*N* = 159).

Time	Variable	Patient		Caregiver		*t*	*p*
		Sum	Average	Sum	Average		
T1	QNN-E	209.62 ± 18.31	4.28 ± 0.37	238.22 ± 11.10	4.86 ± 0.23	−16.846	<0.001
	Enteral access device management	51.01 ± 6.10	4.25 ± 0.51	59.29 ± 2.35	4.94 ± 0.20	−15.974	<0.001
	Therapeutic diet preparation	50.43 ± 5.17	4.20 ± 0.43	58.64 ± 2.90	4.89 ± 0.24	−17.450	<0.001
	Symptom surveillance and complication management	53.88 ± 5.26	4.14 ± 0.40	61.95 ± 5.13	4.77 ± 0.39	−13.841	<0.001
	Longitudinal nutritional counseling	32.09 ± 3.07	4.58 ± 0.44	34.48 ± 1.46	4.93 ± 0.21	−8.859	<0.001
	Adaptive living strategies	22.21 ± 2.82	4.44 ± 0.56	23.87 ± 2.25	4.77 ± 0.45	−5.783	<0.001
T2	QNN-E	202.93 ± 14.88	4.14 ± 0.30	225.74 ± 10.62	4.61 ± 0.22	−15.734	<0.001
	Enteral access device management	47.60 ± 6.21	3.97 ± 0.52	54.04 ± 5.38	4.50 ± 0.45	−9.888	<0.001
	Therapeutic diet preparation	48.20 ± 5.94	4.02 ± 0.49	53.94 ± 5.33	4.50 ± 0.44	−9.071	<0.001
	Symptom surveillance and complication management	52.78 ± 6.05	4.06 ± 0.47	59.65 ± 5.01	4.59 ± 0.39	−11.034	<0.001
	Longitudinal nutritional counseling	32.43 ± 2.00	4.63 ± 0.29	34.78 ± 0.56	4.97 ± 0.08	−14.274	<0.001
	Adaptive living strategies	21.92 ± 1.91	4.38 ± 0.38	23.32 ± 2.15	4.66 ± 0.43	−6.120	<0.001
T3	QNN-E	177.69 ± 16.17	3.63 ± 0.33	199.02 ± 19.19	4.06 ± 0.39	−10.720	<0.001
	Enteral access device management	22.16 ± 14.54	1.85 ± 1.21	37.88 ± 17.94	3.16 ± 1.50	−8.582	<0.001
	Therapeutic diet preparation	55.03 ± 4.47	4.59 ± 0.37	55.23 ± 4.67	4.60 ± 0.39	−0.380	0.704
	Symptom surveillance and complication management	46.86 ± 7.37	3.60 ± 0.57	49.47 ± 5.90	3.81 ± 0.45	−3.487	0.001
	Longitudinal nutritional counseling	30.82 ± 2.31	4.40 ± 0.33	32.79 ± 1.76	4.68 ± 0.25	−8.577	<0.001
	Adaptive living strategies	22.81 ± 1.91	4.56 ± 0.38	23.65 ± 1.99	4.73 ± 0.40	−3.826	<0.001
T4	QNN-E	158.15 ± 10.14	3.23 ± 0.21	165.94 ± 9.51	3.39 ± 0.19	−7.062	<0.001
	Enteral access device management	12.34 ± 2.16	1.03 ± 0.18	12.30 ± 2.93	1.03 ± 0.24	0.153	0.879
	Therapeutic diet preparation	50.31 ± 5.60	4.19 ± 0.47	53.98 ± 4.95	4.50 ± 0.41	−6.199	<0.001
	Symptom surveillance and complication management	43.52 ± 6.65	3.35 ± 0.51	46.00 ± 6.56	3.54 ± 0.50	−3.354	0.001
	Longitudinal nutritional counseling	28.32 ± 2.33	4.05 ± 0.33	29.87 ± 1.83	4.27 ± 0.26	−6.623	<0.001
	Adaptive living strategies	23.68 ± 1.59	4.74 ± 0.32	23.79 ± 1.85	4.76 ± 0.37	−0.553	0.581

T1, Pre-discharge; T2, 2 weeks post-discharge; T3, 1 month post-discharge; T4, 3 months post-discharge; QNN-E, questionnaire of nutritional needs in patients after esophagectomy.

Univariate analyses indicated statistically significant overall differences in QNN-E scores between patients and caregivers across all four time points (T1–T4; all *p* < 0.001; Table 4). Significant variations were also observed among the five nutritional support dimensions at T1 and T2 (all *p* < 0.001). However, no statistically significant differences were detected at specific time points in *Therapeutic Diet Preparation* at T3 (*p* = 0.704) or *Enteral Access Device Management* and *Adaptive Living Strategies* at T4 (*p* = 0.879 and *p* = 0.581, respectively).

### 3.3 Heterogeneous trajectories identified by LCGA

**Figure 4:**
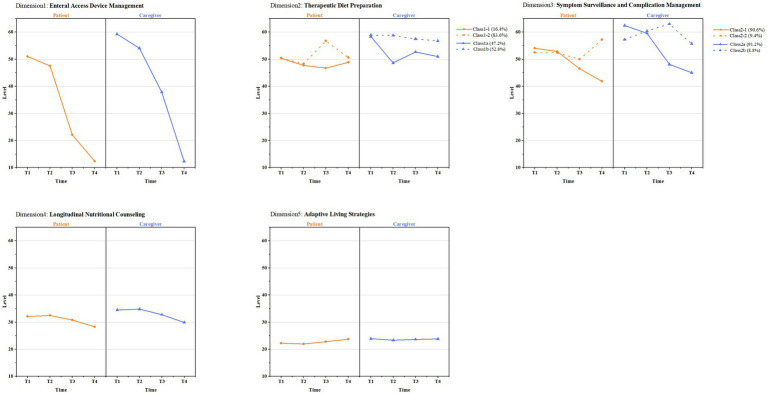
Latent class growth curve of patients and caregivers in 5 dimensions. T1, Pre-discharge; T2, 2 weeks post-discharge; T3, 1 month post-discharge; T4, 3 months post-discharge; Class1-1, consistently stable needs; Class1-2, delayed needs escalation; Class1a, high-need declining; Class1b, high-need sustenance; Class2-1, persistently declining needs; Class2-2, gradually escalating needs; Class2a, rapidly declining high-need; Class2b, gradually increasing low-need.

The nutritional support needs trajectories of the five dimensions were clustered using LCGA (Figure 4; see Supplementary Tables 2–6 for fit statistics, estimated means of the models, and observed individual trajectories). Distinct latent trajectory classes were identified for patients and caregivers within two dimensions: *Therapeutic Diet Preparation* and *Symptom Surveillance and Complication Management*. In the *Therapeutic Diet Preparation* dimension, two distinct patient trajectories emerged: Class 1–1: *Consistently Stable Needs*, this group exhibited a low and stable level of need throughout the observation period. Class 1–2: *Delayed Needs Escalation:* the majority of patients began with moderate needs that remained relatively stable initially but escalated significantly between T3 and T4 (AIC = 3862.615, BIC = 3896.373, aBIC = 3861.552, and entropy = 0.947). Caregiver trajectories in this domain diverged into: Class 1a: *High-need Declining*, caregivers started with very high needs that decreased steadily over time. Class 1b: *High-need Sustenance*, despite some fluctuation, these caregivers maintained persistently high levels of need across all four time points (AIC = 3434.504, BIC = 3468.262, aBIC = 3433.441, and entropy = 0.979). In the *Symptom Surveillance and Complication Management* dimension, patient trajectories were classified as: Class 2–1: *Persistently Declining Needs*, a steady reduction in need from T1 to T4. Class 2–2: *Gradually Escalating Needs*, an initial moderate need that increased progressively, peaking at T4 (AIC = 4135.158, BIC = 4168.916, aBIC = 4134.095, and entropy = 0.890). Caregiver trajectories in this domain showed: Class 2a: *Rapidly Declining High-need*, very high initial need that dropped sharply after discharge. Class 2b: *Gradually Increasing Low-need*, started with low need but showed a concerning upward trend over time (AIC = 3946.276, BIC = 3980.034, aBIC = 3945.213, and entropy = 0. 956).

#### 3.3.1 Univariate analysis of influencing factors of latent class growth trajectories

Univariate analyses identified significant statistical differences in demographic characteristics across distinct latent class growth trajectories for patients and caregivers within the *Therapeutic Diet Preparation* and *Symptom Surveillance and Complication Management* dimensions (Tables 1, 2). In the *Therapeutic Diet Preparation* dimension, enteral access type differed significantly between Class 1–1 (*Consistently Stable Needs*) and Class 1–2 (*Delayed Needs Escalation*) (*p* = 0.007) for patients, whereas education level and relationship with the patient differed significantly between Class 1a (*High-need Declining*) and Class 1b (*High-need Sustenance*) for caregivers (*p* = 0.022 and *p* = 0.005, respectively). In the *Symptom Surveillance and Complication Management* dimension, no significant differences were observed in patients’ demographic characteristics between Class 2–1 (*Persistently Declining Needs*) and Class 2–2 (*Gradually Escalating Needs*). However, for caregivers, the presence of other caregivers differed significantly between Class 2a (*Rapidly Declining High-need*) and Class 2b (*Gradually Increasing Low-need*) (*p* < 0.001).

Independent samples *t*-test were conducted to examine differences in general self-efficacy, QoL, anxiety, depression, BADL, IADL, and nutritional status across latent class trajectories for patients and caregivers (Tables 5, 6). In the *Therapeutic Diet Preparation* dimension, a significant statistical difference was found in patients’ nutritional status between Class 1–1 (*Consistently Stable Needs*) and Class 1–2 (*Delayed Needs Escalation*) (*p* = 0.004). However, no significant difference in general self-efficacy was observed between Class 1a (*High-need Declining*) and Class 1b (*High-need Sustenance*) among caregivers. Within the *Symptom Surveillance and Complication Management* dimension, a significant difference was identified in patients’ BADL between Class 2–1 (*Persistently Declining Needs*) and Class 2–2 (*Gradually Escalating Needs*) (*p* = 0.034). For caregivers, a significant difference in general self-efficacy was found between Class 2a (*Rapidly Declining High-need*) and Class 2b (*Gradually Increasing Low-need*) (*p* = 0.040).

**Table 5:** Comparisons of C-GSES, C-QLQ-OES18, C-SAS, C-SDS, C-BADL, C-IADL, and C-PG-SGA across latent class growth analysis in patients (*N* = 159).

Variable	Total sample, No. (%) (*N* = 159)	Therapeutic diet preparation				Symptom surveillance and complication management			
		Class1-1: consistently stable Needs (*N* = 26)	Class1-2: delayed needs escalation (*N* = 133)	*t*	*p*	Class2-1: persistently declining needs (*N* = 144)	Class2-2: gradually escalating needs (*N* = 15)	*t*	*p*
C-GSES	2.65 ± 0.34	27.65 ± 0.65	29.10 ± 0.33	−1.817	0.071	28.86 ± 0.30	28.87 ± 1.31	−0.004	0.997
C-QLQ-OES18	1.90 ± 1.90	35.38 ± 0.83	35.63 ± 0.31	−0.310	0.757	35.53 ± 0.31	36.20 ± 0.85	−0.668	0.505
C-SAS	1.06 ± 0.22	7.38 ± 0.30	7.41 ± 0.13	−0.087	0.931	7.40 ± 0.13	7.47 ± 0.42	−0.152	0.879
C-SDS	1.26 ± 0.22	8.85 ± 0.34	8.83 ± 0.13	0.034	0.973	8.81 ± 0.12	9.07 ± 0.66	−0.380	0.709
C-BADL	1.56 ± 0.22	9.46 ± 0.24	9.32 ± 0.12	0.517	0.606	9.22 ± 0.20	10.47 ± 0.52	−2.334	0.034
C-IADL	2.48 ± 0.26	19.65 ± 0.38	19.85 ± 0.18	−0.446	0.656	19.73 ± 0.17	20.67 ± 0.37	−1.701	0.091
C-PG-SGA	2.76 ± 0.49	10.04 ± 0.35	11.25 ± 0.17	−2.956	0.004	11.08 ± 0.17	10.73 ± 0.40	0.659	0.511

C-GSES, Chinese version of general self-efficacy scale; C-QLQ-OES18, Chinese version of quality of life questionnaire–oesophageal cancer module; C-SAS, Chinese version of self-rating anxiety scale; C-SDS, Chinese version of self-rating depression scale; C-BADL, Chinese version of basic activities of daily living; C-IADL, Chinese version of instrumental activities of daily living; C-PG-SGA, Chinese version of patient-generated subjective global assessment.

**Table 6:** Comparisons of C-GSES across latent class growth analysis in caregivers (*N* = 159).

Variable	Total sample, No. (%) (*N* = 159)	Therapeutic diet preparation				Symptom surveillance and complication management			
		Class1a: high-need declining (*N* = 75)	Class1b: high-need sustenance (*N* = 84)	*t*	*p*	Class2a: rapidly declining high-need (*N* = 145)	Class2b: gradually increasing low-need (*N* = 14)	*t*	*p*
C-GSES	3.05 ± 0.26	30.56 ± 0.30	30.35 ± 0.29	0.513	0.609	30.58 ± 0.22	29.07 ± 0.65	2.069	0.040

C-GSES, Chinese version of general self-efficacy scale.

#### 3.3.2 Factors associated with the latent class trajectories

Based on the analysis of latent class growth trajectories, in the *Therapeutic Diet Preparation* dimension, significant differences were identified in patients’ enteral access type and nutritional status (PG-SGA score) and caregivers’ education level and relationship to the patient. In the *Symptom Surveillance and Complication Management* dimension, significant differences were observed in patients’ BADLs and availability of other caregivers and general self-efficacy for caregivers.

Binary logistic regression analyses were performed to examine factors associated with trajectory class membership in the *Therapeutic Diet Preparation* and *Symptom Surveillance and Complication Management* models of patients and family caregivers (Tables 7–10). In the *Therapeutic Diet Preparation dimension*, using Class 1–1 (*Consistently Stable Needs*) as the reference group, binary logistic regression analysis revealed that patients with jejunostomy tube placement had significantly higher odds of Class 1–2 (*Delayed Needs Escalation*) membership (*p* = 0.026). Additionally, each unit decrease in nutritional status measured by the PG-SGA was associated with an increased likelihood of Class 1–2 membership (*p* = 0.013). For the *Symptom Surveillance and Complication Management* dimension, with Class 2–1 (*Persistently Declining Needs*) as a reference, poorer BADL performance was significantly associated with a higher probability of Class 2–2 (*Gradually Escalating Needs*) membership (*p* = 0.002).

**Table 7:** Logistic regression analysis examining the association for trajectory of therapeutic diet preparation in Patients.

Variable	Class1-2: delayed needs escalation^a^				
	B	SE	*p*	OR	OR 95%CI
Enteral Access Type (Jejunostomy tube)	1.447	0.648	0.026	4.250	1.193–15.139
C-PG-SGA	0.319	0.129	0.013	1.376	1.069–1.770

C-PG-SGA, Chinese version of patient-generated subjective global assessment.

a Using Class1-1: consistently stable needs as the reference class.

**Table 8:** Logistic regression analysis examining the association for trajectory of symptom surveillance and complication management in patients.

Variable	Class2-2: gradually escalating needs^a^				
	B	SE	*p*	OR	OR 95%CI
C-BADL	0.478	0.154	0.002	1.613	1.192–2.182

C-BADL, Chinese version of basic activities of daily living.

a Using Class2-1: persistently declining needs as the reference class.

**Table 9:** Logistic regression analysis examining the association for trajectory of therapeutic diet preparation in caregivers.

Variable		Class1b: high-need sustenance^a^				
		B	SE	*p*	OR	OR 95%CI
Education level	Junior high school	1.770	1.172	0.131	5.868	0.590–58.410
	Senior high school	2.078	1.190	0.081	7.991	0.776–82.315
	College and/or above	1.448	1.232	0.240	4.256	0.381–47.573
Relationship with patient	Spouse	0.419	0.671	0.532	1.521	0.408–5.664
	Adult child	1.105	0.510	0.030	3.020	1.110–8.212

a Using Class1a: high-need declining as the reference class.

**Table 10:** Logistic regression analysis examining the association for trajectory of symptom surveillance and complication management in caregivers.

Variable	Class2b: gradually increasing low-need^a^				
	B	SE	*p*	OR	OR 95%CI
Other caregivers (No)	3.939	1.062	<0.001	51.384	6.405–412.249
C-GSES	−0.159	0.133	0.231	0.853	0.657–1.107

C-GSES, Chinese version of general self-efficacy scale.

a Using Class2a: rapidly declining high-need as the reference class.

In the *Therapeutic Diet Preparation* dimension, family caregivers who were adult children of the patient were significantly more likely than those in other relational roles (e.g., spouse or other relatives) to belong to Class 1b (*High-need Sustenance*) compared with Class 1a (*High-need Declining*) as the reference group (*p* = 0.030). In the *Symptom Surveillance and Complication Management* dimension, with Class 2a (*Rapidly Declining High-need*) as a reference, the absence of additional caregivers was strongly associated with Class 2b (*Gradually Increasing Low-need*) membership (*p* < 0.001).

## 4 Discussion

This prospective longitudinal study provides novel insights into the dynamic and heterogeneous nature of nutritional support needs within the patient–caregiver dyad during the critical three-month period following radical esophagectomy. While overall nutritional care needs declined from pre-discharge (T1) to 3 months post-discharge (T4), LCGA uncovered distinct subpopulations with divergent pathways, particularly in the domains of *Therapeutic Diet Preparation* and *Symptom Surveillance and Complication Management*. This finding moves beyond describing a population-average trend and underscores the critical need for personalized, trajectory-informed care strategies.

In the *Therapeutic Diet Preparation* dimension, the majority of patients (83.6%) followed a *Delayed Needs Escalation* trajectory (Class 1–2), characterized by initially lower support needs that increased significantly by the third post-discharge month. This pattern was independently associated with the presence of a jejunostomy tube and poorer nutritional status at baseline. A plausible explanation is that patients discharged with enteral feeding tubes experience a prolonged phase of formula-based nutrition, which may defers the complex cognitive and practical demands of transitioning to an oral therapeutic diet (34). Consequently, their need for detailed dietary guidance surges later during the oral intake resumption phase, highlighting a potential window of vulnerability where standard early post-discharge education may be inadequate (11). Conversely, caregivers in this domain were nearly evenly distributed between *High-need Declining* (Class 1a, 47.2%) and *High-need Sustenance* (Class 1b, 52.8%) trajectories. Adult children of the patient and those with higher educational levels were more likely to belong to the sustained high-need group. This may reflect the dual pressures faced by the “sandwich generation,” who must simultaneously balance caregiving with career and their own potential family obligations, leading to persistent anxiety and a continuous need for accessible, actionable information to manage dietary complexity (35, 36).

Within the *Symptom Surveillance and Complication Management* dimension, while most patients (90.6%) showed *Persistently Declining Needs*, a small but important subgroup (9.4%) exhibited a *Gradually Escalating Needs* trajectory (Class 2–2). This escalation was significantly linked to poorer baseline performance in BADLs, suggesting that functional limitations may impair patients’ ability to self-monitor and manage symptoms, leading to increasing uncertainty and support needs over time. For caregivers, higher general self-efficacy and the presence of additional family caregivers were associated with the *Rapidly Declining High-need* trajectory (Class 2a). This findings imply that robust personal coping resources and shared caregiving responsibilities may facilitate adaptation and mastery of symptom management tasks, allowing their perceived needs to decrease more rapidly (37).

These findings advocate for a paradigm shift from a uniform, time-based post-discharge support model to a dynamic, trajectory-informed, and dyad-centered approach. Clinical interventions should be tailored not only to the postoperative timepoint but also to the specific risk profile of the patient-caregiver dyads. To operationalize this approach, future research should develop and evaluate integrated online management platforms or eHealth systems. Such systems could continuously monitor dyads’ evolving needs through periodic digital assessments, automatically identify those entering high-risk trajectories (e.g., *Delayed Needs Escalation*), and deliver timely, tailored educational content, reminders, and professional support. This would enable proactive, precision support aligned with the actual longitudinal needs of each dyad.

For patients at high risk of delayed dietary challenges (e.g., those with a jejunostomy tube, poorer PG-SGA scores), nutritional counseling should be strategically intensified beyond the immediate discharge period. Proactive, scheduled follow-ups around the anticipated time of tube removal and diet advancement (e.g., weeks 6–12) are crucial to address the escalating needs identified in our data. These considerations, however, warrant further testing in prospective longitudinal studies.

For caregivers in the *High-need Sustenance* trajectory, particularly adult children, support should be designed for efficiency and clarity. This group may benefit from structured, digital health tools (e.g., mobile apps with reminder systems, video demonstrations) and concise, evidence-based guidelines that accommodate their busy schedules and demands for just-in-time information (38). Moreover, assessing and bolstering caregiver self-efficacy—especially for those without additional support—should be a key component of early discharge planning, as it appears to be an important protective factor for managing symptom-related distress (13).

This study reinforces the family caregivers’ role as both proxies for unmet patient needs and independent clients with their own burden. The observed association between sustained high caregiver needs in critical domains (e.g., *therapeutic diet preparation*, *symptom surveillance and complication management*) and increased psychological strain demonstrates that supporting the caregiver is a fundamental component of achieving positive patient outcomes. Therefore, interventions should adopt a family-centered framework, providing skills-based training (e.g., enteral device management, complication recognition) alongside psychosocial support to alleviate burden (13, 39). For families with multiple caregivers, collective education sessions at discharge can ensure shared understanding and task coordination (37).

### 4.1 Limitations

This study had several limitations. First, despite an adequate overall sample size, some of the identified latent trajectory classes were relatively small (e.g., the ‘Gradually Escalating Needs’ class among patients comprised only 9.4%). This class imbalance may limit the stability of estimates for these specific subgroups and reduce the statistical power to detect their associated factors. Second, while we adjusted for key baseline variables, residual confounding by unmeasured factors (e.g., nuanced social support dynamics, specific postoperative complication types, or healthcare access) remains possible, which is an inherent limitation of observational designs. Third, owing to the limited time points for assessment data collection, the LCGA only converged with a strong parametric assumption. This simplification may have obscured more nuanced trajectory patterns of need evolution. For example, variables such as the adoption of new eating habits could not be incorporated into the logistic regression analyses due to temporal constraints. Furthermore, it remains unknown whether new changes in *Therapeutic Diet Preparation* dimension will emerge among different patient trajectories as they adapt to long-term oral feeding. Future studies incorporating more frequent longitudinal assessments would help capture richer and more complex nutritional support trajectories. Fourth, the generalizability of the findings may be limited by the modest sample size and single-center study design. The participants were recruited from a single tertiary hospital, which may not be representative of the broader population of patients undergoing esophagectomy. Consequently, the identified trajectories and their associated factors should be interpreted with caution. Future multicenter studies with larger sample sizes are warranted to validate and extend these results.

## 5 Conclusion

In conclusion, this study identified distinct, heterogeneous trajectories of nutritional support needs in esophagectomy patient-caregiver dyads during the critical three-month post-discharge period. Key factors such as enteral access type, patient nutritional and functional status, caregiver relationship and education level, and caregiver self-efficacy predict membership in more vulnerable trajectory classes. These findings provide a robust evidence base for transitioning from uniform, reactive post-discharge care toward a precision supportive care model that is stratified, proactive, and dyad-centered. Tailored interventions—timed to anticipated inflection points in need trajectories and matched to individual risk profiles—hold promise for improving nutritional outcomes, reducing caregiver burden, and enhancing overall recovery for both patients and their essential care partners.

## Data Availability

The original contributions presented in the study are included in the article/supplementary material, further inquiries can be directed to the corresponding author/s.

## Glossary

QoL: quality of life
NRS 2002: Nutritional risk screening 2002
T1: pre-discharge
T2: 2 weeks post-discharge
T3: 1 month post-discharge
T4: 3 months post-discharge
QNN-E: the questionnaire of nutritional needs in patients after esophagectomy
C-GSES: Chinese general self-efficacy scale
C-QLQ-OES18: Chinese quality of life questionnaire–oesophageal cancer module
C-HADS: Chinese hospital anxiety and depression scale
C-ADL: Chinese activities of daily living scale
C-PG-SGA: Chinese patient-generated subjective global assessment
LGCM: latent growth curve model
LCGA: latent class growth analysis
*df*: degrees of freedom
RMSEA: root mean square error of approximation
SRMR: standardized root mean square residual
CFI: comparative fit index
AIC: Akaike information criteria
BIC: Bayesian information criteria
aBIC: adjusted BIC
LRT: likelihood ratio test
BLRT: bootstrap likelihood ratio test
