# Supplementary material for: Heterogeneous trajectories of nutritional support needs in patient–caregiver dyads following radical esophagectomy: a latent class growth analysis
Source: Front Nutr. 2026 May 14;13:1789423. doi: 10.3389/fnut.2026.1789423 (PMC13215927; doi:10.3389/fnut.2026.1789423)
Supplement: Supplementary file 1 [file Table_1.docx]

eTable 1 Latent growth curve model results and fitting indicators.

| Participant | Model | χ^2^/*df* | *p* | CFI | RMSEA | SRMR | Mean | | | Variance | | |
| --- | --- | --- | --- | --- | --- | --- | --- | --- | --- | --- | --- | --- |
|  |  |  |  |  |  |  | Intercept | Slope | Curve Slope | Intercept | Slope | Curve Slope |
| Patient | Linear | 3.089 | 0.005 | 0.000 | 0.345 | 0.587 | 207.113*** | -8.014*** |  | -8.421 | 6.831** |  |
|  | Nonlinear | 3.089 | 0.005 | 0.000 | 0.546 | 0.431 | 213.802*** | -19.069*** | 1.620*** | -74.153 | -71.101 | -2.380 |
|  | Free Estimation | 3.089 | 0.005 | 0.882 | 0.056 | 0.055 | 209.903*** | -7.204*** |  | 12.797 | 1.637 |  |
| Caregiver | Linear | 11.532 | ＜0.001 | 0.000 | 0.323 | 0.420 | 236.588*** | -11.592*** |  | 0.774 | 5.754*** |  |
|  | Nonlinear | 11.532 | ＜0.001 | 0.275 | 0.537 | 0.373 | 240.708*** | -19.507*** | 1.192*** | -82.300* | -86.097 | -1.237 |
|  | Free Estimation | 11.532 | ＜0.001 | 1.000 | 0.000 | 0.062 | 238.260*** | -12.559*** |  | 5.751 | 3.362** |  |

**p*＜0.05, ***p*＜0.01, ****p*＜0.001

eTable2-1 Latent class growth analysis results and fitting indicators in Enteral Access Device Management.

| Participant | *N* of classes | K | AIC | BIC | aBIC | Entropy | LMR | BLRT | Class Probability |
| --- | --- | --- | --- | --- | --- | --- | --- | --- | --- |
| Patient | 1 | 8 | 4068.690 | 4093.241 | 4067.917 |  |  |  |  |
|  | 2 | 11 | 3576.851 | 3610.609 | 3575.788 | 1.000 | 0.7346 | 0.000 | 0.975/0.025 |
|  | 3 | 14 | 3581.078 | 3624.043 | 3579.725 | 0.753 | 0.6143 | 0.5176 | 0.025/0.044/0.931 |
|  | 4 | 17 | 3588.851 | 3641.023 | 3587.208 | 1.000 | 0.3858 | 1.000 | 0.975/0/0/0.025 |
| Caregiver | 1 | 8 | 3882.912 | 3907.463 | 3882.139 |  |  |  |  |
|  | 2 | 11 | 3542.532 | 3576.290 | 3541.468 | 1.000 | 0.515 | 0.000 | 0.994/0.006 |
|  | 3 | 14 | 3353.697 | 3396.662 | 3352.344 | 0.999 | 0.105 | 0.000 | 0.006/0.943/0.050 |
|  | 4 | 17 | 3359.697 | 3411.869 | 3358.054 | 0.998 | 0.618 | 1.000 | 0.943/0.050/0.006/0.000 |

eTable2-2 Latent class growth analysis results and fitting indicators in Enteral Access Device Management.

| Participant | Class | Parameter (Mean) | | Estimate | Standard Error | *p* |
| --- | --- | --- | --- | --- | --- | --- |
| Patient | Class1 | Mean | Intercept | 51.013 | 0.482 | ＜0.001 |
|  |  |  | Slope | -3.415 | 0.680 | ＜0.001 |
| Caregiver | Class1 | Mean | Intercept | 59.289 | 0.186 | ＜0.001 |
|  |  |  | Slope | -5.252 | 0.489 | ＜0.001 |

eTable3-1 Latent class growth analysis results and fitting indicators in Therapeutic Diet Preparation.

| Participant | *N* of classes | K | AIC | BIC | aBIC | Entropy | LMR | BLRT | Class Probability |
| --- | --- | --- | --- | --- | --- | --- | --- | --- | --- |
| Patient | 1 | 8 | 3930.272 | 3954.824 | 3929.499 |  |  |  |  |
|  | 2 | 11 | 3862.615 | 3896.373 | 3861.552 | 0.947 | 0.000 | 0.000 | 0.164/0.836 |
|  | 3 | 14 | 3844.905 | 3887.870 | 3843.552 | 0.886 | 0.000 | 0.000 | 0.648/0.195/0.157 |
|  | 4 | 17 | 3842.951 | 3895.122 | 3841.308 | 0.787 | 0.208 | 0.095 | 0.572/0.088/0.157/0.182 |
| Caregiver | 1 | 8 | 3686.570 | 3711.121 | 3685.797 |  |  |  |  |
|  | 2 | 11 | 3434.504 | 3468.262 | 3433.441 | 0.979 | 0.000 | 0.000 | 0.472/0.528 |
|  | 3 | 14 | 3363.951 | 3406.916 | 3362.598 | 0.974 | 0.241 | 0.000 | 0.478/0.075/0.447 |
|  | 4 | 17 | 3302.964 | 3355.136 | 3301.321 | 0.950 | 0.229 | 0.000 | 0.075/0.340/0.484/0.101 |

eTable3-2 Latent class growth analysis results and fitting indicators in Therapeutic Diet Preparation.

| Participant | Class | Parameter | | Estimate | Standard Error | *p* |
| --- | --- | --- | --- | --- | --- | --- |
| Patient | Class1 | Mean | Intercept | 48.805 | 0.628 | ＜0.001 |
|  |  |  | Slope | 0.618 | 0.257 | 0.016 |
|  | Class2 | Mean | Intercept | 50.349 | 0.461 | ＜0.001 |
|  |  |  | Slope | -1.951 | 0.707 | 0.006 |
| Caregiver | Class1 | Mean | Intercept | 58.345 | 0.355 | ＜0.001 |
|  |  |  | Slope | -9.699 | 0.370 | ＜0.001 |
|  | Class2 | Mean | Intercept | 58.488 | 0.363 | ＜0.001 |
|  |  |  | Slope | 0.018 | 0.441 | 0.967 |

eTable4-1 Latent class growth analysis results and fitting indicators in Symptom Surveillance and Complication Management.

| Participant | *N* of classes | K | AIC | BIC | aBIC | Entropy | LMR | BLRT | Class Probability |
| --- | --- | --- | --- | --- | --- | --- | --- | --- | --- |
| Patient | 1 | 8 | 4155.023 | 4179.574 | 4154.250 |  |  |  |  |
|  | 2 | 11 | 4135.158 | 4168.916 | 4134.095 | 0.890 | 0.001 | 0.000 | 0.906/0.094 |
|  | 3 | 14 | 4126.673 | 4169.638 | 4125.320 | 0.781 | 0.072 | 0.000 | 0.447/0.075/0.478 |
|  | 4 | 17 | 4118.469 | 4170.640 | 4116.826 | 0.850 | 0.015 | 0.000 | 0.088/0.069/0.421/0.421 |
| Caregiver | 1 | 8 | 4011.803 | 4036.354 | 4011.030 |  |  |  |  |
|  | 2 | 11 | 3946.276 | 3980.034 | 3945.213 | 0.956 | 0.005 | 0.000 | 0.912/0.088 |
|  | 3 | 14 | 3901.148 | 3944.112 | 3899.794 | 0.909 | 0.097 | 0.000 | 0.088/0.094/0.818 |
|  | 4 | 17 | 3851.663 | 3903.834 | 3850.020 | 0.966 | 0.324 | 0.000 | 0.151/0.057/0.044/0.748 |

eTable4-2 Latent class growth analysis results and fitting indicators in Symptom Surveillance and Complication Management.

| Participant | Class | Parameter | | Estimate | Standard Error | *p* |
| --- | --- | --- | --- | --- | --- | --- |
| Patient | Class1 | Mean | Intercept | 54.018 | 0.459 | ＜0.001 |
|  |  |  | Slope | -1.201 | 0.667 | 0.072 |
|  | Class2 | Mean | Intercept | 51.735 | 0.920 | ＜0.001 |
|  |  |  | Slope | 0.437 | 0.271 | 0.107 |
| Caregiver | Class1 | Mean | Intercept | 62.441 | 0.405 | ＜0.001 |
|  |  |  | Slope | -2.878 | 0.582 | ＜0.001 |
|  | Class2 | Mean | Intercept | 58.927 | 1.453 | ＜0.001 |
|  |  |  | Slope | 0.296 | 0.386 | 0.443 |

eTable5-1 Latent class growth analysis results and fitting indicators in Longitudinal Nutritional Counseling.

| Participant | *N* of classes | K | AIC | BIC | aBIC | Entropy | LMR | BLRT | Class Probability |
| --- | --- | --- | --- | --- | --- | --- | --- | --- | --- |
| Patient | 1 | 8 | 2929.162 | 2953.713 | 2928.388 |  |  |  |  |
|  | 2 | 11 | 2925.330 | 2959.088 | 2924.267 | 0.967 | 0.289 | 0.035 | 0.019/0.981 |
|  | 3 | 14 | 2917.409 | 2960.374 | 2916.056 | 0.868 | 0.143 | 0.000 | 0.642/0.220/0.138 |
|  | 4 | 17 | 2917.136 | 2969.308 | 2915.493 | 0.889 | 0.107 | 0.129 | 0.635/0.120/0.025/0.220 |
| Caregiver | 1 | 8 | 2123.473 | 2148.024 | 2122.700 |  |  |  |  |
|  | 2 | 11 | 1949.371 | 1983.129 | 1948.308 | 1.000 | 0.035 | 0.000 | 0.151/0.849 |
|  | 3 | 14 | 1922.159 | 1965.124 | 1920.806 | 0.990 | 0.013 | 0.000 | 0.025/0.151/0.824 |
|  | 4 | 17 | 1909.554 | 1961.725 | 1907.911 | 0.953 | 0.364 | 0.000 | 0.761/0.063/0.145/0.031 |

eTable5-2 Latent class growth analysis results and fitting indicators in Longitudinal Nutritional Counseling.

| Participant | Class | Parameter | | Estimate | Standard Error | *p* |
| --- | --- | --- | --- | --- | --- | --- |
| Patient | Class1 | Mean | Intercept | 32.088 | 0.243 | ＜0.001 |
|  |  |  | Slope | 0.340 | 0.287 | 0.236 |
| Caregiver | Class1 | Mean | Intercept | 34.478 | 0.116 | ＜0.001 |
|  |  |  | Slope | 0.302 | 0.126 | 0.017 |

eTable6-1 Latent class growth analysis results and fitting indicators in Adaptive Living Strategies.

| Participant | *N* of classes | K | AIC | BIC | aBIC | Entropy | LMR | BLRT | Class Probability |
| --- | --- | --- | --- | --- | --- | --- | --- | --- | --- |
| Patient | 1 | 8 | 2706.519 | 2731.070 | 2705.746 |  |  |  |  |
|  | 2 | 11 | 2629.846 | 2663.604 | 2628.783 | 0.971 | 0.007 | 0.000 | 0.082/0.918 |
|  | 3 | 14 | 2610.258 | 2653.222 | 2608.904 | 0.962 | 0.001 | 0.000 | 0.874/0.038/0.088 |
|  | 4 | 17 | 2578.183 | 2630.355 | 2576.540 | 0.996 | 0.445 | 0.000 | 0.151/0.176/0.296/0.377 |
| Caregiver | 1 | 8 | 2731.078 | 2755.629 | 2730.305 |  |  |  |  |
|  | 2 | 11 | 2212.415 | 2246.173 | 2211.352 | 1.000 | 0.000 | 0.000 | 0.258/0.742 |
|  | 3 | 14 | 2111.076 | 2154.041 | 2109.723 | 0.997 | 0.344 | 0.000 | 0.239/0.113/0.648 |
|  | 4 | 17 | 1978.861 | 2031.033 | 1977.218 | 1.000 | 0.345 | 0.000 | 0.604/0.107/0.151/0.138 |

eTable6-2 Latent class growth analysis results and fitting indicators in Adaptive Living Strategies.

| Participant | Class | Parameter | | Estimate | Standard Error | *p* |
| --- | --- | --- | --- | --- | --- | --- |
| Patient | Class1 | Mean | Intercept | 22.214 | 0.223 | ＜0.001 |
|  |  |  | Slope | -0.289 | 0.265 | 0.276 |
| Caregiver | Class1 | Mean | Intercept | 23.868 | 0.177 | ＜0.001 |
|  |  |  | Slope | -0.547 | 0.218 | 0.012 |
